# Factors influencing parents’ healthcare-seeking behaviour for their ill children: a cross-sectional study based on China family panel studies

**DOI:** 10.3389/fpubh.2026.1839390

**Published:** 2026-06-22

**Authors:** Shuqi Xu, Chunxia Miao, Yun Zhao, Huanhuan Jia

**Affiliations:** School of Management, Xuzhou Medical University, Xuzhou, Jiangsu, China

**Keywords:** child health, healthcare, healthcare services, health-seeking behaviour, public health

## Abstract

**Background:**

Parents’ healthcare-seeking behaviour during their children’s illnesses plays a vital role in ensuring their healthy development. This study conducted a systematic analysis of the familial and societal factors affecting child health, with the intention of providing evidence-based insights to enhance child health outcomes and improve the efficiency of healthcare service delivery.

**Methods:**

Using data from the China Family Panel Studies (CFPS) 2022 survey, variables such as children’s medical insurance status, parental educational level, per capita household income, and place of residence were selected. The analysis was conducted using independent-samples *t*-tests, χ^2^ tests, and multivariable binary logistic regression analyses to investigate the patterns and influencing factors associated with parents’ healthcare-seeking behaviour when their children became ill.

**Results:**

A total of 5,361 subjects were included, with male subjects accounting for 53.48% of the sample. Among the respondents, 43.84% (*n* = 2,350) adopted informal health-seeking (IHS) behaviour and 56.15% (*n* = 3,011) adopted prompt formal health-seeking (PFHS) behaviour. A univariate analysis revealed statistically significant differences in healthcare-seeking behaviour across sex and family economic status. A multivariate analysis further revealed child age, paternal education, geographic region, and urban–rural residence as independent influencing factors. In the univariate analysis, a tendency toward informal health-seeking behaviour was observed with increasing child age, higher parental education, and higher family income.

**Conclusion:**

In this study, parents’ healthcare-seeking behaviour for their ill children was significantly influenced by a variety of socioeconomic factors, including parental education level, child age, and region of residence. These findings underscore the importance of guiding families toward appropriate healthcare choices, which is crucial for optimising child health outcomes and increasing the efficiency of public health service delivery.

## 1 Introduction

Common childhood diseases, including acute fever, diarrhoea, and influenza, constitute a significant public health burden worldwide, not only affecting the healthy development of children but also imposing a heavy economic strain on families (1, 2). Fortunately, the clinical management of common childhood diseases is generally well standardised. When children receive timely, effective, and safe treatment, their symptoms can be alleviated and the associated economic burden on families can be reduced.

As the primary guardians of minors, parents play a key role in managing their children’s health (3, 4). Parental attitudes and behaviours not only serve as models for children to emulate but also directly influence child health outcomes through health-related decisions (5). However, significant disparities exist in the level of importance parents place on child health and their responses to their children’s illnesses. Studies have shown that, in the Pacific region (6), each one-level increase in parental education is associated with a 1.5% reduction in the risk of child hospitalisation due to illness and a 25% increase in the likelihood of choosing private hospitalisation. This finding reflects that parents with higher education levels are more inclined to protect their children’s health through preventive medical investments. In addition, a family’s financial resources influence their participation in children’s medical insurance. Children from high-income families are more likely to possess both commercial and basic health insurance, and a father’s higher level of education is associated with a stronger willingness to purchase insurance (7). Moreover, there are substantial disparities between urban and rural areas. Reports in China (8) have revealed a significant gap in infant and under-five mortality rates between urban and rural areas. In 2020, the rural infant mortality rate was 5.4%, and the rural under-five mortality rate was 7.5%—both higher than their urban counterparts. In rural areas, the probability of parents seeking medical treatment for their children decreases with an increase in the number of children. Indeed, the probability of seeking medical treatment for only one child was 0.112; however, it decreased to 0.085 when the number of children exceeded three (9). These findings indicate that parents with different family structures, places of residence, education levels, and economic conditions devote varying levels of attention to children’s health and demonstrate different responses to illness.

In addition, parents sometimes delay seeking medical treatment or adopt inappropriate self-treatment, such as irrational medication use, which may aggravate their children’s conditions and lead to serious consequences. Research has indicated that, for children with chronic kidney disease, parents who recognize symptoms in a timely manner and actively seek medical care can facilitate timely treatment and significantly reduce the risks of complications and renal failure. Conversely, delays in diagnosis and treatment often result in difficult-to-treat conditions and avoidable mortality (10). Researchers in Somaliland (11) have reported that, on average, each child experiences 8.4 episodes of avoidable illnesses caused by delayed medical care, with children and their families suffering and losing more because they do not receive timely and effective treatment. Therefore, differences in parents’ health literacy, educational backgrounds, and family economic conditions may influence healthcare-seeking decisions and, consequently, contribute to adverse health outcomes for their children. This issue warrants further discussion.

In China, the rapid development of the medical and health system coexists with continuing regional differences and inequality in parents’ health literacy, resulting in significant variations in how parents manage common childhood illnesses. Existing research in China (9) has indicated that the level of parental education has a significant impact on children’s health and that higher levels of parental education are associated with better child health outcomes. Parents with different educational backgrounds in urban and rural areas also demonstrate differences in child vaccination behaviours and access to health information, which in turn affects the prevention and control of infectious diseases (12). Given that the early childhood development stage has profound implications for long-term health, systematically examining the level of importance parents place on child health and its influencing factors is particularly crucial.

Based on this background, this study aimed to systematically analyse the impact mechanisms of China’s urban–rural disparities, parental education levels, family economic status, and health investment behaviours on child health and to identify key influencing factors in order to provide a theoretical basis and policy guidance for optimising healthcare service utilisation and improving child health outcomes.

## 2 Materials and methods

### 2.1 Data resources

The China Family Panel Studies (CFPS), administered by the Institute of Social Science Survey (ISSS) at Peking University, constitutes a large-scale, nationwide, multidisciplinary longitudinal social survey. This project aims to capture the dynamics of China’s social, economic, demographic, educational, and health transformations through the tracking of individuals, households, and communities. It serves as a critical data infrastructure for academic research and public policy analysis. CFPS survey data are of high quality and have been widely used in various studies.

The CFPS employs four core instruments: Community questionnaires, Household questionnaires, Adult questionnaires, and Child questionnaires. Overtime, these have evolved into specialised instruments, including long-form/short-form versions, proxy-administered questionnaires, and computer-assisted telephone interview modules tailored to diverse family structures. This study used a proxy-administered child questionnaire to assess child-specific indicators, adult questionnaires to extract parental demographic characteristics, and household questionnaires to determine socioeconomic status.

### 2.2 Selection and measurement of variables

The respondents in this study were asked, “*What is the most common action you take when your child has a minor illness (such as fever or diarrhoea)*?” The response options were as follows: (1) seek medical consultation immediately, (2) self-medicate/purchase medication, (3) use traditional folk remedies (such as Gua Sha), (4) seek divine intervention/perform rituals, or (5) take no action and await natural recovery.

The dichotomy of parental healthcare-seeking behaviour into prompt formal health-seeking (PFHS) behaviour and informal health-seeking (IHS) behaviour is grounded in the health behaviour model and the pathway to care model. According to the health belief model and pathway-to-care model, care-seeking decisions are fundamentally a choice between professional medical services and lay/self-management (13, 14). In the context of childhood minor illnesses, self-medication, traditional remedies, and watchful waiting represent conceptually equivalent alternatives to formal care—they all lack professional diagnosis and treatment planning. Several large-scale studies (15, 16) in low- and middle-income countries have adopted this identical dichotomisation, as it directly addresses the public health question of whether families utilise formal healthcare when a child becomes ill. Therefore, while this study acknowledges non-trivial differences among informal strategies, collapsing them into a single IHS category is methodologically justified for the specific research aim of comparing formal versus informal care utilization. Following data cleaning, the responses to this question were categorised for analysis: Option (1) was classified as PFHS behaviour. All other options (2)–(5) were collectively classified as IHS behaviour.

Additional covariates included characteristics of the children and their parents. The child characteristics included sex (male/female), age (continuous variable), medical insurance status (such as none, social health insurance or commercial health insurance only, or dual coverage), number of medical visits, and total medical expenses due to illness over the past 12 months (continuous variables). The parental characteristics included the highest level of education attained (such as primary school or below, junior high school, senior high/vocational school, associate degree, or bachelor’s degree). The household characteristics included per capita annual income (in RMB), place of residence (such as urban or rural), and geographic region (such as Eastern China, Western China, Central China, and Northeast China).

### 2.3 Data analysis

Statistical analyses were performed using Stata/SE 16.0, with statistical significance defined as a two-tailed *p*-value of <0.05. Descriptive statistics characterised the cohort using continuous variables expressed as the mean ± standard deviation (normally distributed) or the median with interquartile range (non-normally distributed), while categorical variables were summarised as frequencies and percentages. Univariate comparisons between the PFHS and IHS groups employed independent-samples *t*-tests for child age (normally distributed), χ^2^ tests for categorical variables (such as sex, medical insurance, parental education, place of residence, and region), and Mann–Whitney *U* tests for per capita household income (non-normally distributed). In addition, Mann–Whitney *U* tests were performed for skewed healthcare utilization metrics (such as number of medical visits and total health expenditure). A multivariable binary logistic regression analysis was used to model PFHS adoption, incorporating all predictors.

## 3 Results

### 3.1 Study population characteristics

A total of 5,361 subjects were included in the study. The mean age of the children of the surveyed parents was 8.1 ± 4.1 years, with the majority being male (53.5%) (Table 1). The majority of children (72.4%) were covered by either social medical insurance or commercial health insurance, while dual coverage was observed in 18.4% of the cases. Regarding parental education, 57.6% of fathers and 59.9% of mothers had attained a junior high school education or lower. Only 11.5% of fathers and 12.5% of mothers held bachelor’s degrees or higher. The median per capita household income was RMB 18,542, with the upper quartile at RMB 31,500 and the lower quartile at RMB 11,111. Geographically, 52.5% of the families resided in urban areas, while 47.5% resided in rural settings. The participants were predominantly from Eastern China (32.5%) and Western China (31.8%).

**Table 1:** Comparison of child, parental, and household characteristics between informal health-seeking (IHS, *n* = 2,350) and prompt formal health-seeking (PFHS, *n* = 3,011) behaviour groups.

Variables		Overall *n*(%)	Adopted approach		χ2/I-test/*z*	*P-*value
			Informal health-seeking behaviour	Prompt formal health-seeking behaviour		
Age (mean ± SD)		8.11 ± 4.13	8.40 ± 4.03	7.89 ± 4.19	4.478	<0.001
Per capita household income (Median)		18542.86	20000.00	17333.33	−6.559	<0.001
Sex	Male	2,867 (53.48)	1,252 (43.67)	1,615 (56.33)	0.069	0.973
	Girls	2,494 (46.52)	1,098 (44.03)	1,396 (55.97)		
Medical insurance	Uninsured	490 (9.14)	203 (41.43)	287 (58.57)	7.062	0.029
	SMI or CHI	3,883 (72.43)	1,678 (43.21)	2,205 (56.79)		
	Dual coverage	988 (18.43)	469 (47.47)	519 (52.53)		
Parental education	Primary or below	1,057 (19.72)	441 (41.72)	616 (58.28)	27.624	<0.001
	Junior high	2029 (37.85)	844 (41.60)	1,185 (58.40)		
	Senior high/vocational	1,029 (19.19)	441 (42.86)	588 (57.14)		
	Associate degree	632 (11.79)	306 (48.42)	326 (51.58)		
	Bachelor+	614 (11.45)	318 (51.79)	296 (48.21)		
Maternal education	Primary or below	1,206 (22.50)	495 (41.04)	711 (58.96)	26.496	<0.001
	Junior high	2004 (37.38)	845 (42.17)	1,159 (57.83)		
	Senior high/vocational	839 (15.65)	365 (43.50)	474 (56.50)		
	Associate degree	644 (12.01)	296 (45.96)	348 (54.04)		
	Bachelor+	668 (12.46)	349 (52.25)	319 (47.75)		
Place of residence	Rural	2,548 (47.53)	980 (38.46)	1,568 (61.54)	56.953	<0.001
	Urban	2,813 (52.47)	1,370 (48.70)	1,443 (51.30)		
Region	Eastern China	1743 (32.51)	777 (44.58)	966 (55.42)	147.705	<0.001
	Central China	1,527 (28.48)	524 (34.32)	1,003 (65.68)		
	Western China	1707 (31.84)	790 (46.28)	917 (53.72)		
	Northeast China	384 (7.16)	259 (67.45)	125 (32.55)		
Overall		5,361(100)	2,350 (43.84)	3,011 (56.16)		

SMI, social medical insurance; CHI, commercial health insurance.

### 3.2 Differences in parental approaches

A total of 56.2% (*n* = 3,011) of the respondents adopted PFHS for minor childhood illnesses, while 43.8% (*n* = 2,350) chose informal approaches (Table 1). Significant differences existed between groups. PFHS was more common among families with younger children (*t* = 4.478; *p* < 0.001). Parents also adopt different approaches when they have different types of insurance. Families with uninsured children had higher PFHS rates (χ^2^ = 7.062; *p* = 0.029). With respect to parental education, PFHS decreased with higher paternal education (χ^2^ = 27.624; *p* < 0.001). Maternal education showed a similar trend (χ^2^ = 26.496, *p* < 0.001). The per capita household income in the PFHS group was lower than that in the IHS group (*z* = −6.559, *p* < 0.001). In addition, Northeast China had the lowest PFHS rate (χ^2^ = 147.705, *p* < 0.001), and rural residents preferred PFHS (χ^2^ = 56.953, *p* < 0.001). Finally, no sex difference was observed (*p* = 0.973).

### 3.3 Healthcare utilisation and expenditure of different adopted approaches

This study compares resource use between groups (Table 2). PFHS was associated with higher median number of visits (*p* < 0.001). Median health expenditure was 50% higher in the PFHS group (*p* < 0.001).

**Table 2:** Multivariable binary logistic regression results for PFHS (versus IHS).

Variables	Adopted approach M (P25, P75)		*U*	*z*	*P*-value
	Informal health-seeking behaviour (*n* = 2,350)	Prompt formal health-seeking behaviour (*n* = 3,011)			
Number of medical visits	0 (0, 2)	1 (0, 3)	2660951.5	−15.624	<0.001
Total healthcare expenditure	200 (0, 925)	300 (50, 1,000)	3230956.0	−5.432	<0.001

Comparison of healthcare utilisation and expenditure between informal health-seeking behaviour and prompt formal health-seeking behaviour groups.

### 3.4 Multivariable predictors of health-seeking behaviour

A binary logistic regression analysis revealed significant independent predictors of PFHS after adjusting for covariates (Table 3). Child age demonstrated a substantive inverse relationship with PFHS, whereby each additional year of age reduced the likelihood of PFHS by 4.4% (*p* < 0.001). Paternal education exhibited a paradoxical gradient: compared with fathers holding primary education or less, those with associate degrees had 26% lower PFHS odds (*p* = 0.005), while bachelor’s degree holders showed an even stronger reduction of 32% (*p* = 0.001). Geographic disparities were particularly striking, with Central China residents demonstrating 49% higher PFHS odds than Eastern China counterparts (*p* < 0.001), whereas Western and Northeast China showed significantly reduced adoption (*p* = 0.009 and *p* < 0.001, respectively). Residential setting further modified outcomes, with urban dwellers having 32% lower PFHS odds than their rural counterparts (*p* < 0.001). Notably, maternal education, child sex, per capita household income, and insurance status did not retain statistical significance in the multivariable model.

**Table 3:** Factors associated with the adopted approach when children are ill.

Variables		B	SE	Wald χ2	*P*-value	Exp(B)	95% CI
Age		−0.045	0.007	40.122	<0.001	0.956	(0.942, 0.969)
Father’s education	Primary or below						
	Junior high	−0.017	0.080	0.046	0.831	0.983	(0.841, 1.149)
	Senior high/vocational	−0.120	0.093	1.641	0.200	0.887	(0.739, 1.065)
	Associate degree	−0.303	0.109	7.773	0.005	0.738	(0.596, 0.914)
	Bachelor+	−0.386	0.112	11.868	0.001	0.680	(0.546, 0.847)
Region	Eastern China						
	Central China	0.401	0.074	29.809	<0.001	1.494	(1.293, 1.725)
	Western China	−0.187	0.071	6.925	0.009	0.829	(0.721, 0.953)
	Northeast China	−0.969	0.121	64.4	<0.001	0.380	(0.300, 0.481)
Living place	Rural						
	Urban	−0.388	0.060	41.036	<0.001	0.679	(0.603, 0.764)
Constant		0.951	0.108	77.389	<0.001	2.587	

## 4 Discussion

Using data from the CFPS, this study systematically examined the multidimensional factors that influence parental health decision-making when children were ill. By constructing a multivariable binary logistic regression model, this study identified and verified the role of factors such as children’s insurance participation, parental education level, per capita household annual income, and urban versus rural residence in shaping parents’ decision-making regarding medical treatment. Only four factors remained significant independent predictors: child age, paternal education, geographic region, and residential setting. It is worth emphasising that per capita household annual income, maternal education level, and child insurance status showed significant crude associations with PFHS in univariate analyses, but none remained statistically significant after the multivariable binary logistic regression analysis. This finding revealed that these factors are not independent predictors of health-seeking behaviour; rather, their apparent effects are largely explained by confounding factors—most likely paternal education, child age, and urban–rural residence. Furthermore, the research revealed these factors’ pathways of influence on child health outcomes. The results provide an empirical basis for further understanding the mechanisms underlying child health inequality and provide a useful reference for promoting fair access to children’s health services and for optimising public health policies.

The multivariable analysis revealed that paternal education level was a significant independent predictor of parents’ health-seeking behaviour, whereas maternal education level was not. This result is consistent with findings from a study in Zimbabwe, which suggested that fathers’ education affects the health outcomes of under-five children and is more influential than that of mothers (17). Parents with higher education levels in the household tended to adopt IHS when their children became ill, which may be related to their higher health literacy and greater information acquisition capacity. Research has indicated that education not only directly enhances health cognition but also improves an individual’s capacity for health management through indirect pathways, such as by increasing the efficiency of internet use and optimising health behaviours (18). Highly educated parents are typically more proactive in acquiring, discerning, and applying health knowledge, leading them to be more confident in employing informal management strategies in certain situations. Therefore, highly educated parents may have more confidence in their children’s self-care. However, this finding differs from that of Wamani et al. (19), who found that the mother’s education level, rather than the father’s education level, was a predictor of child health inequalities in rural Uganda. Studies in Indonesia (20) have also shown that young children are more influenced by maternal education. These two seemingly contradictory conclusions may be due to cultural differences. In the traditional Chinese Confucian culture system, patriarchy has a strong influence, and the father, as the head of the family, controls the distribution and decision-making power of family resources. Thus, the role of the father has a more comprehensive and profound impact on the growth and health of children.

With respect to child age, parents demonstrated a stronger preference for PFHS when younger children became ill, and the proportion of parents who purchased medication for self-treatment increased as children grew older. Child age demonstrated a substantive and independent inverse relationship with PFHS. This trend is consistent with findings from a study in northwestern Tanzania, which reported a morbidity prevalence of 26.1% among under-five children, decreasing to 10.4% in the 15–19-year age group (21). As children age, their immunity strengthens, and the incidence of common illnesses decreases, consequently reducing parental anxiety and leading to a greater inclination toward IHS. The Ethiopian study (22) also provides support from the perspective of maternal experience: as mothers age, their ability to recognize childhood illnesses and manage health issues improves, and they are more likely to self-manage their children’s common ailments. It is generally well-known that young children with immature immune systems are at higher risk of infection. Studies in Indonesia (23) have further demonstrated that parents are more likely to seek professional medical care to reduce health risks.

Regarding regional differences, rural residents in this study were more likely to choose PFHS when their children were sick. This phenomenon may be related to the continuous improvement of the medical security system in rural China. In recent years, policies such as the New Cooperative Medical Scheme (NCMS) have expanded health insurance coverage for the rural population, enhancing both the willingness and the ability to utilise formal medical services. A study on adult health service utilisation in China showed that urban residents are 3.24 times more likely to choose informal health services than rural residents (24), reflecting progress in the accessibility of formal healthcare in rural areas. A Malaysian study showed that the prevalence of sickness among adults in rural areas was higher than that among urban adults (25). A similar pattern may exist among children, suggesting that the tendency for rural residents to choose PFHS may be due to more severe illness conditions. Conversely, urban residents, who are more likely to experience minor illnesses and opt for IHS, may not necessarily have poorer access to care. In addition, this study found that parents residing in Central China were more inclined to choose formal healthcare services for their children than those in Eastern and Western China. This difference may stem from imbalances in socioeconomic development levels, climatic environments, and the allocation of medical resources across regions, with socioeconomic factors being identified as a key variable influencing healthcare choices (26).

Parents tend to adopt PFHS when their children are ill, reflecting the importance they attach to their children’s health and their trust in the health system. Notably, many common childhood diseases may cause serious complications, and even death, that can be effectively prevented when they are treated properly at an early stage (27). Therefore, parents’ active choice of formal medical services may be associated with better child health outcomes and improved disease prognosis.

Notably, this theoretical association is complicated by potential reverse causality and confounding. A study from Nigeria revealed that 12.2% of children receiving IHS had severe illness, compared with 10.7% of children receiving PFHS (28). While such an association between PFHS and lower rates of severe illness has been observed in previous studies, it must be interpreted with caution due to inherent limitations of cross-sectional study designs. Specifically, our cross-sectional design cannot determine whether receiving PFHS leads to better clinical outcomes or whether seeking PFHS simply identifies children with more severe underlying illness. The higher healthcare utilization and expenditure observed in the PFHS group may therefore partially reflect greater baseline disease severity rather than a causal effect of formal care itself. Based on these findings, this study recommends that public health campaigns, while encouraging the utilisation of formal services, should also target groups such as highly educated parents by providing clear, evidence-based self-care guidelines and warning signs. This may guide in making appropriate decisions on when to seek professional care, while avoiding delays in treating severe conditions due to overconfidence. Moreover, policymakers should continue to consolidate and improve the medical security system in rural areas, effectively reducing the economic barriers faced by low-income families and ensuring that children have equitable access to timely and effective medical services.

This study grouped self-medication, traditional remedies, and no action into a single IHS category to contrast with PFHS. This study acknowledges that this lumping masks heterogeneity within IHS; for example, self-medication for a mild fever is qualitatively different from complete inaction for a high fever. However, this grouping was intentional and theory-driven: the research question concerns the binary decision of whether parents seek formal care versus any form of informal management. Moreover, prior studies have shown that even seemingly benign informal strategies (such as over-the-counter medication) can delay appropriate care if warning signs are missed (11). Importantly, the main finding—that higher parental education and income are associated with greater IHS—is unlikely to be reversed by disaggregation. In fact, *post-hoc* inspection of our data (not shown as a separate analysis) suggests that self-medication dominates IHS among higher socioeconomic groups, while no action is rare across all groups. Therefore, the observed IHS tendency among advantaged families primarily reflects a preference for self-management rather than neglect. Readers should interpret the IHS category as a proxy for “non-professional management” rather than uniformly inappropriate care. Future studies with dedicated sample sizes may further explore predictors of each informal subtype.

Several limitations warrant consideration. First, parents reported their “most common action” for minor childhood illnesses, reflecting a general tendency rather than a specific episode. While this reduces recall bias, it may introduce social desirability bias (overreporting of formal care). Any non-differential misclassification would likely bias associations toward the null, making our significant findings conservative. Second, the dichotomous classification of PFHS versus IHS inevitably involves some misclassification (such as parents who typically self-medicate may occasionally seek formal care), but such non-differential error would not systematically favour our observed patterns. Third, the cross-sectional design precludes causal inference. Although the direction from education and income to care-seeking is theoretically clear, we cannot rule out unmeasured confounding (such as health literacy and prior morbidity) or reverse causality (such as frequent illness affecting income). Therefore, results should be interpreted as associations, not causal effects. Prospective studies are needed to establish temporality. Fourth, this study lacked data on illness severity at the time of the care-seeking decision. Severity is a likely confounder: children with more severe symptoms are more likely to receive prompt formal care and incur higher expenses. Therefore, the observed associations between PFHS and higher healthcare utilisation/expenditure should not be interpreted as evidence that formal care causes higher resource use.

## 5 Conclusion

Based on data from the CFPS, this study systematically analysed parental healthcare-seeking decisions and their influencing factors when children fell ill. The findings indicated that higher parental education levels and older child age were associated with a greater tendency to use informal healthcare services. Conversely, residence in rural areas predisposed parents to adopt prompt formal healthcare services. Notably, while factors such as maternal education, child sex, per capita household income, and insurance status showed significant crude associations in univariate analyses, they did not retain statistical significance in the multivariable model.

Based on the findings of this study, tailored recommendations are proposed for specific subgroups. (1) For older children, parents should be guided to recognize that minor symptoms can be managed with appropriate self-care. Persistent high fever or respiratory distress warrants prompt formal healthcare-seeking behaviour. This is particularly important because older children may present with atypical symptoms, which can delay the recognition of serious illness. (2) For highly educated parents, who in this study demonstrated a greater tendency to choose IHS—it is essential to distinguish between appropriate self-care for self-limiting conditions and dangerous delays in seeking care. Guidance should be provided to prevent overconfidence in home management. (3) For urban populations, where IHS was more common, public health messages should emphasise that convenience does not replace clinical safety. Urban parents should be encouraged to use formal services when severe symptoms are present. (4) For regions with lower PFHS uptake, interventions should focus on reducing structural barriers while simultaneously educating parents to recognise that delays in seeking formal care for certain conditions may lead to preventable complications or death.

Beyond China, these findings have broader implications. Several other developing countries, particularly in South and Southeast Asia, Latin America, and globally, also face similar challenges of urban–rural disparities, health insurance coverage gaps, and varying parental health literacy. In this study, the tendency toward IHS behaviours among highly educated parents and the rural disadvantage in accessing formal healthcare are likely generalisable to these regions. This study has proposed specific recommendations for different subgroups. These recommendations provide a transferable framework for designing targeted child health interventions globally.

This research sheds light on the intrinsic connections between family socioeconomic characteristics and child health service utilisation, while also highlighting the pivotal role of parental decision-making in child health management. The results enrich the understanding of the mechanisms underlying child health inequalities, provide new empirical evidence for understanding household health behaviours, offer insights for optimising the allocation of child healthcare resources and improving the accessibility of primary care services, and contribute to the development of tailored health promotion strategies for families with different socioeconomic backgrounds.

## Data Availability

The datasets presented in this study can be found in online repositories. The names of the repository/repositories and accession number(s) can be found at: https://www.isss.pku.edu.cn/cfps/en/.
